# Performance of PCR/Electrospray Ionization-Mass Spectrometry on Whole Blood for Detection of Bloodstream Microorganisms in Patients with Suspected Sepsis

**DOI:** 10.1128/JCM.01860-19

**Published:** 2020-08-24

**Authors:** Kristoffer Strålin, Richard E. Rothman, Volkan Özenci, Kieron Barkataki, David Brealey, Neelam Dhiman, Lara Poling, Michael C. Kurz, Ajit P. Limaye, Frank LoVecchio, Kristin Lowery, Loren G. Miller, Gregory J. Moran, J. Scott Overcash, Amisha Parekh, W. Frank Peacock, Emanuel P. Rivers, Matthew Sims, Amy M. Stubbs, Martin Sundqvist, Måns Ullberg, Karen C. Carroll

**Affiliations:** a Department of Infectious Diseases, Karolinska University Hospital, Stockholm, Sweden; b Department of Medicine Huddinge, Karolinska Institutet, Stockholm, Sweden; c Department of Emergency Medicine, Johns Hopkins University School of Medicine, Baltimore, Maryland, USA; d Department of Clinical Microbiology, Karolinska University Hospital, Stockholm, Sweden; e Division of Clinical Microbiology, Department of Laboratory Medicine, Karolinska Institutet, Stockholm, Sweden; f Department of Emergency Medicine, Kern Medical Center, Bakersfield, California, USA; g Division of Critical Care, NIHR Biomedical Research Centre at University of College London Hospitals NHS Foundation Trust, London, United Kingdom; h Molecular Infectious Diseases, med fusion/Quest Diagnostics, Lewisville, Texas, USA; i AthoGen Testing, Carlsbad, California, USA; j Department of Emergency Medicine, University of Alabama School of Medicine, Birmingham, Alabama, USA; k University of Washington, Seattle, Washington, USA; l University of Arizona, Maricopa Medical Center, Phoenix, Arizona, USA; m Lundquist Institute at Harbor-UCLA Medical Center, Torrance, California, USA; n Department of Emergency Medicine, Olive View-UCLA Medical Center, Sylmar, California, USA; o eStudySite, Sharp Chula Vista, San Diego, California, USA; p Department of Emergency Medicine, New York Methodist Hospital, New York, New York, USA; q Baylor College of Medicine, Houston, Texas, USA; r Department of Emergency Medicine and Surgery, Henry Ford Hospital, Detroit, Michigan, USA; s Department of Surgery, Henry Ford Hospital, Detroit, Michigan, USA; t Beaumont Health, Royal Oak and Oakland University William Beaumont School of Medicine, Rochester, Michigan, USA; u Department of Emergency Medicine, Truman Medical Center, University of Missouri—Kansas City, Kansas City, Missouri, USA; v Department of Laboratory Medicine, Clinical Microbiology, Faculty of Medicine and Health, Örebro University, Örebro, Sweden; w Division of Medical Microbiology, Department of Pathology, The Johns Hopkins University School of Medicine, Baltimore, Maryland, USA; Boston Children's Hospital

**Keywords:** sepsis, bacteremia, direct detection, PCR/ESI-MS

## Abstract

Blood culture (BC) often fails to detect bloodstream microorganisms in sepsis. However, molecular diagnostics hold great potential. The molecular method PCR/electrospray ionization-mass spectrometry (PCR/ESI-MS) can detect DNA from hundreds of different microorganisms in whole blood. The aim of the present study was to evaluate the performance of this method in a multicenter study including 16 teaching hospitals in the United States (*n* = 13) and Europe (*n* = 3). First, on testing of 2,754 contrived whole blood samples, with or without spiked microorganisms, PCR/ESI-MS produced 99.1% true-positive and 97.2% true-negative results. Second, among 1,460 patients with suspected sepsis (sepsis-2 definition), BC and PCR/ESI-MS on whole blood were positive in 14.6% and 25.6% of cases, respectively, with the following result combinations: BC positive and PCR/ESI-MS negative, 4.3%; BC positive and PCR/ESI-MS positive, 10.3%; BC negative and PCR/ESI-MS positive, 15.3%; and BC negative and PCR/ESI-MS negative, 70.1%. Compared with BC, PCR/ESI-MS showed the following sensitivities (coagulase-negative staphylococci not included): Gram-positive bacteria, 58%; Gram-negative bacteria, 78%; and *Candida* species, 83%. The specificities were >94% for all individual species. Patients who had received prior antimicrobial medications (*n* = 603) had significantly higher PCR/ESI-MS positivity rates than patients without prior antimicrobial treatment—31% versus 22% (*P* < 0.0001)—with pronounced differences for Gram-negative bacteria and *Candida* species. In conclusion, PCR/ESI-MS showed excellent performance on contrived samples. On clinical samples, it showed high specificities, moderately high sensitivities for Gram-negative bacteria and *Candida* species, and elevated positivity rates during antimicrobial treatment. These promising results encourage further development of molecular diagnostics to be used with whole blood for detection of bloodstream microorganisms in sepsis.

## Abstract (precis)

Blood culture (BC) often fails to detect bloodstream microorganisms in sepsis. However, molecular diagnostics hold great potential. The molecular method PCR/electrospray ionization-mass spectrometry (PCR/ESI-MS) can detect DNA from hundreds of different microorganisms in whole blood. The aim of the present study was to evaluate the performance of this method in a multicenter study including 16 teaching hospitals in the United States (*n* = 13) and Europe (*n* = 3).

## INTRODUCTION

The World Health Organization (WHO) recently recognized sepsis as a global health priority, as it is a common and severe disease that can often be cured with adequate treatment, including appropriate antimicrobial therapy (1, 2). In order to enable targeted antimicrobial therapy with maximum effect and avoid unnecessary use of broad-spectrum antimicrobials, the microbiological diagnosis of sepsis should be established (3). However, even in patients with known bacterial sepsis, blood culture (BC) often provides negative results (4). For improved detection of bloodstream pathogens, a number of commercial molecular methods have been developed (5). Unfortunately, most methods are limited by a narrow spectrum of detectable microorganisms (e.g., the T2Bacteria panel [T2 Biosystems]) (6) or suboptimal sensitivity (e.g., the LightCycler SeptiFast test [Roche]) or specificity (e.g., the Magicplex sepsis real-time test [Seegene] and the Karius test [Karius]) (5, 7).

Based on the PCR/electrospray ionization-mass spectrometry (PCR/ESI-MS) technology, Abbott (Carlsbad, CA) developed the IRIDICA BAC BSI assay, which has the capacity to detect DNA from >200 different microorganisms in whole-blood samples (8). Clinical diagnostic studies have shown promising results, with PCR/ESI-MS-positive detections typically exceeding BC-positive results (9, 10). However, the previous studies of PCR/ESI-MS on whole blood have been too small to enable evaluation on individual microorganisms and to compare the performance of the method on samples from patients with and without prior antimicrobial medication (11).

The aims of the present study were (i) to test PCR/ESI-MS on blood samples spiked with known microorganisms (contrived specimens) and (ii) to compare PCR/ESI-MS with BC on whole blood from patients with suspected sepsis in a large multicenter study. The study was the basis for an application to the U.S. Food and Drug Administration (FDA) regarding the IRIDICA BAC BSI assay. However, Abbott withdrew the FDA application and ceased producing IRIDICA instruments and IRIDICA test kits in 2017.

## MATERIALS AND METHODS

### Study design and settings.

This was a prospective, multicenter, observational cohort study, with patients enrolled and samples collected from December 2014 through March 2016 at 16 teaching hospitals in three countries: United States (*n* = 13; Baylor College of Medicine, Houston, TX; Harbor-UCLA Medical Center, Torrance, CA; Henry Ford Hospital, Detroit, MI; Johns Hopkins Hospital, Baltimore, MD; Kern Medical Center, Bakersfield, CA; Maricopa Medical Center, Phoenix, AZ; New York Methodist Hospital, New York, NY; Olive View-UCLA Medical Center, Sylmar, CA; eStudySite, Sharp Chula Vista, San Diego, CA; Truman Medical Center, Kansas City, MO; Beaumont Hospital, Royal Oak, MI; University of Alabama, Birmingham, AL; and University of Washington, Seattle, WA); Sweden (*n* = 2; Karolinska University Hospital, Stockholm, and Örebro University, Örebro); and the United Kingdom (*n* = 1; University College London Hospitals, London).

The study also included four clinical testing sites, each with an installed IRIDICA PCR/ESI-MS system, that included Johns Hopkins Hospital, Baltimore, MD, USA; Karolinska University Hospital, Stockholm, Sweden; med fusion, Lewisville, TX, USA; and AthoGen Testing, Carlsbad, CA, USA.

Clinical whole-blood samples from enrolled study patients were collected and stored at −70°C and later sent to the clinical testing sites for analysis by PCR/ESI-MS (IRIDICA BAC BSI). In addition, the clinical testing sites also analyzed contrived specimens with PCR/ESI-MS.

### Contrived whole-blood specimens.

EDTA-containing whole-blood lots were collected by Ibis Biosciences (Abbott) from 110 healthy adults, 500 ml from each subject. The whole-blood lots were prescreened for contaminating bacterial DNA using the IRIDICA BAC BSI assay, and contaminated lots were excluded. Each whole-blood lot was split into aliquots of 5 ml that were spiked with culture-quantified stocks of 50 different microorganisms (see Table S1 in the supplemental material).

For each microorganism, the limit of detection (LOD) was determined. Whole-blood aliquots were spiked with microorganisms at 3 to 10 different concentrations (5 samples at each concentration). The lowest concentrations for which all samples were PCR/ESI-MS positive were then used in a confirmation analysis of additionally 20 spiked samples. The confirmed LOD was defined as the lowest concentration (in CFU per milliliter) for which the detection rate was at least 95% (minimum of 19/20 valid replicates). In Table S1, the confirmed LOD of 50 microorganisms are presented.

It is well known that the concentration of bacteria in the bloodstream varies among patients with bloodstream infection (12). Thus, in order to reflect a patient scenario with different bloodstream concentrations of microorganisms, whole-blood aliquots were spiked to the following target levels: 1.5× LOD (25 aliquots), 3× LOD (15 aliquots), and 10× LOD (10 aliquots). Altogether, 50 contrived blood samples of each of 50 microorganisms were made, totaling 2,500 specimens.

In addition, from the prescreened EDTA whole-blood lots from healthy adults described above, Ibis Biosciences provided 254 specimens without spiked microorganisms (negative contrived specimens).

### Patients.

Patients aged ≥6 years who presented to the emergency department or who were being cared for in the hospital’s intensive care unit (ICU) or other similar units with suspected sepsis according to the sepsis-2 definition, i.e., suspected bloodstream infection and a diagnosis of systemic inflammatory response syndrome (SIRS) (13), motivating standard-of-care BC, were eligible for inclusion. The SIRS diagnosis required at least two of the following SIRS criteria: body temperature of >38°C or < 36°C, heart rate of >90 beats/minute, respiratory rate of >20/min or a partial CO_2_ pressure of <32 mm Hg, and white blood cell count of >12,000 cells/μl or <4,000 cells/μl. The single exclusion criterion was previous enrollment in the study. Data on antimicrobial medication taken within 14 days prior to enrollment were collected from each patient’s record by chart review shortly after enrollment.

From each study patient, at least 10 ml whole blood was collected in 1 or 2 EDTA tubes for testing with PCR/ESI-MS, concurrently with standard-of-care BC.

### PCR/ESI-MS.

PCR/ESI-MS (IRIDICA BAC BSI) was performed at the clinical testing sites according to the manufacturer's instructions. The assay was designed to identify unique DNA sequences from >200 different bacteria and fungi for species-level identification, as well as the antibiotic resistance markers *mecA*, *vanA*, *vanB*, and *bla*_KPC_. A negative control was included in every run, and a positive control was included at least once per day of analysis. Four different positive controls, supplied by ZeptoMetrix Corporation (Buffalo, NY), were used on a rotating basis, i.e., whole-blood samples spiked with either methicillin-resistant Staphylococcus aureus (MRSA) bundled with Candida albicans, vancomycin-resistant Enterococcus faecium (VRE), vancomycin-resistant Enterococcus faecalis, or carbapenem-resistant Klebsiella pneumoniae (KPC). The analytic procedure was run in two separate rooms, one room for sample preparation and DNA extraction and the other room for PCR, desalting, and mass spectrometry. Assay turnaround time was approximately 8 h, and system throughput was 5 patient samples at a time, permitting a maximum of 15 samples per 24 h. Operating the IRIDICA system required one full-time laboratory technologist.

Briefly, 5 ml of whole blood was lysed using the IRIDICA bead beater. DNA was extracted with the IRIDICA DNA preparation kit, using the automated extraction system. Purified DNA in buffer was automatically distributed by the IRIDICA sample preparation into 16-well IRIDICA BAC BSI assay strips containing PCR reagents and primers for 18 PCRs. PCR was performed on the IRIDICA thermal cycler using a preloaded PCR amplification protocol. After PCR amplification, the IRIDICA BAC BSI assay strips were loaded onto the IRIDICA desalter, which purified DNA to remove substances that might interfere with mass spectrometry. Following desalting, plates were loaded onto the IRIDICA mass spectrometer. Purified amplicons were injected one well at a time into an electrospray ionization time-of-flight mass spectrometer for determination of the molecular mass of the amplicons. The resulting information was used for species identification by automated database comparison, as previously described (8).

The IRIDICA BAC BSI assay strip contained an internal control template at a known concentration, which generated a control amplicon. The ratio of the amount of the amplicon in the sample DNA to that of the control amplicon was reported as a “level,” which represented a semiquantitative marker of the DNA content of the sample.

### Blood cultures.

BC was carried out as standard of care. One or two sets of BC bottles were collected, each set consisting of one aerobic and one anaerobic bottle. The standardized and accredited blood culture systems of each study hospital were used. Identification and susceptibility testing of the species were performed according to the local laboratory standards, including matrix-assisted laser desorption ionization–time-of-flight mass spectrometry, VITEK2 (bioMérieux, Durham, NC, USA), and disc diffusion and Etest gradient diffusion. No information about blood volume in the BC bottles was available.

### Statistics.

IBM SPSS Statistics (20.0) software was used for statistical analyses. Chi-square and Fisher´s exact tests were used for comparison of proportions, and the Mann-Whitney U test was used for comparison of independent groups. A *P* value of <0.05 was considered significant.

### Ethics.

The study was approved by an ethical board at each study site and was conducted according to the requirements of the individual country’s laws and regulations and the Declaration of Helsinki. All study participants provided written informed consent.

## RESULTS

### Limits of detection and contrived specimens.

Table S1 shows the confirmed LOD for individual microorganisms. There was no significant difference between the LOD of Gram-positive and Gram-negative bacteria, with a median of 48 CFU/ml (interquartile range [IQR], 16 to 128 CFU/ml) versus a median of 32 CFU/ml (IQR, 16 to 64 CFU/ml) (*P* = 0.24). However, the LOD of *Candida* species, with a median of 8 CFU/ml (IQR, 6 to 12 CFU/ml), were significantly lower than those of Gram-positive (*P* = 0.012) and Gram-negative (*P* = 0.016) bacteria. It should be noted that coagulase-negative staphylococci (CoNS) had high LOD; e.g., that for Staphylococcus epidermidis was 256 CFU/ml.

The results of PCR/ESI-MS for 2,500 positive contrived specimens are shown in Table S2. PCR/ESI-MS identified the inoculated organism (true-positive result) in 2,477 cases (99.1%) and detected other organisms (false positives) in 33 cases (1.3%). The false-positive results included Cutibacterium acnes (*n* = 7), Nocardia farcinica (*n* = 4), Escherichia coli (*n* = 3), S. aureus (*n* = 3), S. epidermidis (*n* = 3), and 9 other species with 1 or 2 positive results each (see Table S2).

Among 254 negative contrived specimens, the PCR/ESI-MS system reported true-negative results in 247 cases (97.2%). False-positive results were noted in 7 cases, one each of *C. acnes*, *Nocardia* species, E. coli, S. aureus, Staphylococcus lugdunensis, *Micrococcus* species, and *Mycobacterium* species.

There were 255 contrived samples with microorganisms with known resistance markers (143 with *mecA*, 35 with *vanA*, 43 with *vanB*, and 34 with *bla*_KPC_). All of these resistance markers were correctly detected by PCR/ESI-MS. Among 1,143 contrived samples spiked with microorganisms known not to harbor any of the four resistance markers, there were 4 false positives for *mecA* (0.3%) but no false positives for the other resistance markers.

### Patients.

**FIG 1:**
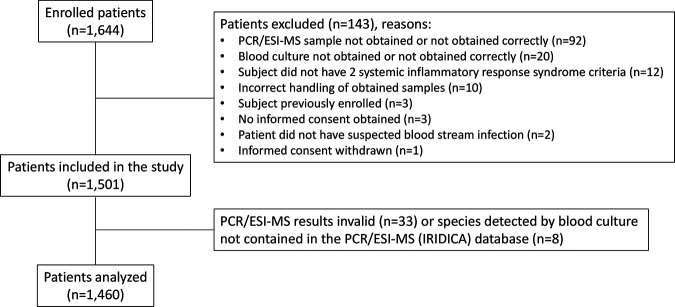
Flow chart of study population.

Altogether, 1,501 patients were included in the study (Fig. 1). They had a median age of 54 years (range, 6 to 96 years), and 625 patients (41.6%) were females.

Forty-one patients had PCR/ESI-MS results that were either invalid (*n* = 33) or not comparable with BC results (*n* = 8), meaning that the microorganisms reported by BC were not part of the PCR/ESI-MS organism reporting list. These patients were omitted from the study, and thus, the results for 1,460 patients were used in the final analyses. Two sets of BC bottles were obtained for 995 patients (68.2%), and one set was obtained for 465 patients (31.8%).

Among 1,460 study patients, 603 patients (41.3%) had received any antimicrobial medication within 14 days prior to enrollment (antibiotics in 555 patients, antifungals in 79 patients, and antivirals in 114 patients).

### Results of blood culture and PCR/ESI-MS in patients and clinical samples.

**FIG 2:**
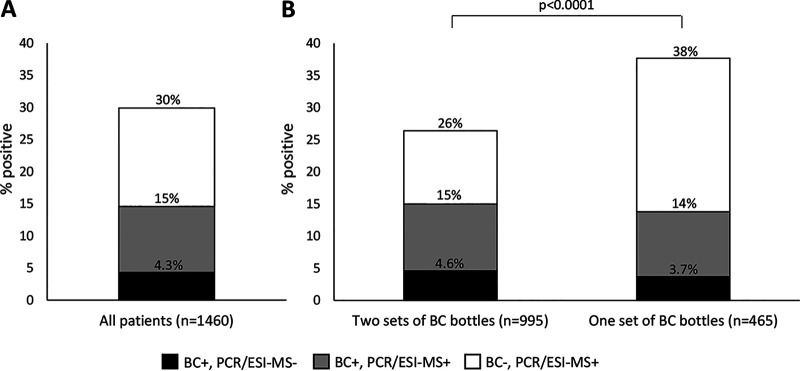
Proportion of patients positive by BC and/or PCR/ESI-MS altogether (A) and among patients with two sets and one set of BC bottles (B).

In the study group of 1,460 patients with suspected sepsis, a microorganism was detected by either BC or PCR/ESI-MS or both in 437 patients (29.9%), i.e., by BC in 213 patients (14.6%) and by PCR/ESI-MS in 374 patients (25.6%). The following result combinations were noted: BC positive and PCR/ESI-MS negative (*n* = 63), BC positive and PCR/ESI-MS positive (*n* = 150), and BC negative and PCR/ESI-MS positive (*n* = 224) (Fig. 2A). Table 1 shows the combined results of PCR/ESI-MS and BC. Concordant negative results were noted for 1,023 patients. Fully concordant positive results (identical specimens detected by BC and PCR/ESI-MS) were noted for 113 patients, of whom 109 had concordant single microorganisms and 4 had concordant multiple microorganisms. Among 150 BC-positive PCR/ESI-MS-positive patients, fully discordant results (different species detected by BC and PCR/ESI-MS) were noted in 8 cases (5.3%). Figure 2B shows combined positive results of BC and PCR/ESI-MS among patients with two sets of BC bottles and among those with one set of BC bottles. As noted, the BC positivity rate was similar between the two categories of patients (15% and 14%), but BC positivity and PCR/ESI-MS positivity combined were significantly more common among patients with one set of BC bottles.

**TABLE 1:** Combined numbers of organisms detected by PCR/ESI-MS and BC in patients with suspected sepsis

No. of organisms detected by:		No. (%) of patients (*n* = 1,460)
PCR/ESI-MS	BC	
0	0	1,023 (70)
0	1	53 (3.6)
0	≥2	10 (0.7)
1	0	187 (13)
1	1	114^*a*^ (7.8)
1	≥2	8^*b*^ (0.5)
≥2	0	37 (2.5)
≥2	1	18^*c*^ (1.2)
≥2	≥2	10^*d*^ (0.7)

a The same organism was detected by PCR/ESI-MS and BC in 109/114 cases. Four patients were PCR/ESI-MS positive for Escherichia coli and BC positive for coagulase-negative staphylococci. One patient was PCR/ESI-MS positive for *Candia albicans* and BC positive for *Bacteroides* species.

b The organism detected by PCR/ESI-MS was also detected by BC in all 8 cases.

c The organism detected by BC was also detected by PCR/ESI-MS in 15/18 cases. One patient was BC positive for *Peptostreptococcus* species and PCR/ESI-MS positive for *Bacteroides* species and *Clostridium* species; one patient was BC positive for Eggerthella lenta and PCR/ESI-MS positive for *Bacteroides* species and *Fusobacterium* species; and one patient was BC positive for Streptococcus anginosus and PCR/ESI-MS positive for Gemella morbillorum and two anaerobic bacterial species.

d The identical organisms were detected by PCR/ESI-MS and BC in 4/10 cases. At least one organism was detected by both PCR/ESI-MS and BC in 10/10 cases.

Among 25 patients with positive BC for CoNS with two sets of BC bottles analyzed, CoNS was detected in both BCs in 8 cases and in just one BC in 17 cases.

**FIG 3:**
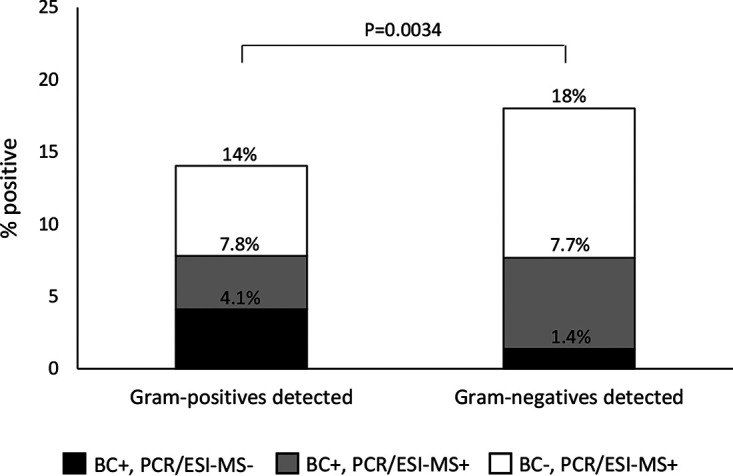
Proportion of patients positive by BC and/or PCR/ESI-MS for Gram-positive and Gram-negative bacteria.

**FIG 4:**
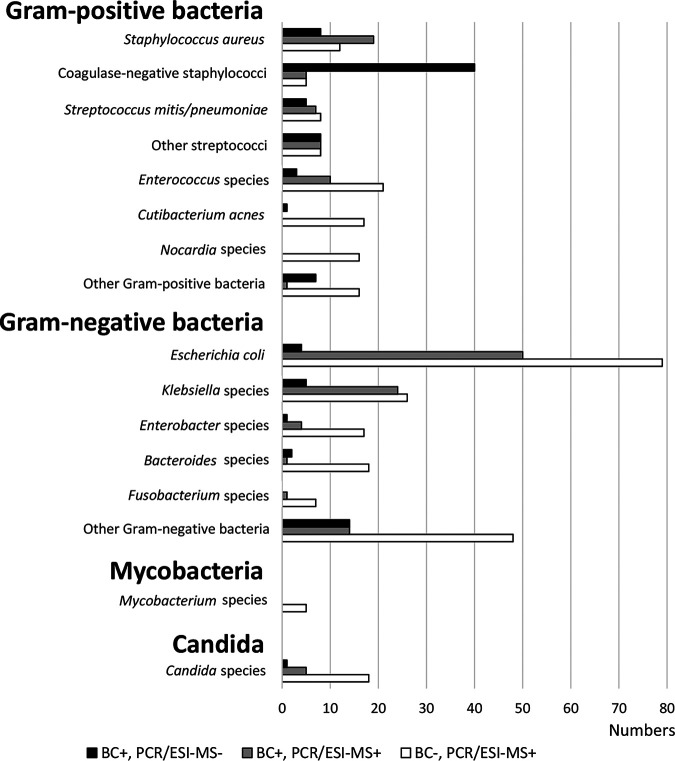
Individual organisms detected by BC and PCR/ESI-MS.

Figure 3 shows the combined positive results of BC and PCR/ESI-MS for Gram-positive and Gram-negative bacteria. As noted, the positive BC rate was similar between Gram-positive (7.8%) and Gram-negative (7.7%) bacteria. However, as noted in Fig. 3, the combination of BC positivity and PCR/ESI-MS positivity was significantly more common for Gram-negative than for Gram-positive bacteria. In addition, PCR/ESI-MS positivity was significantly more common for Gram-negative than for Gram-positive bacteria, 243/1,460 (16.6%) versus 145/1,460 (9.9%) (*P* < 0.0001). The same pattern was noted for individual microorganisms (Fig. 4 and Table S3).

All non-S. aureus staphylococci observed in the study were categorized as CoNS and included S. epidermidis, Staphylococcus hominis, Staphylococcus capitis, and Staphylococcus haemolyticus. CoNS were detected by BC in 45 patients (3.1%) and by PCR/ESI-MS in 10 patients (0.68%) (*P* < 0.0001) (Fig. 4).

*Candida* species were detected by BC in 6 patients (0.41%) and by PCR/ESI-MS in 23 patients (1.6%) (*P* = 0.0028) (Fig. 4).

### Sensitivities and specificities of PCR/ESI-MS compared with blood culture.

When results for individual species were considered (CoNS not included), the sensitivity of PCR/ESI-MS compared with BC was 71% (144/203) overall, 58% (45/77) for Gram-positive bacteria, 78% (94/120) for Gram-negative bacteria, and 83% (5/6) for *Candida* species. The specificities were >94% for all individual species. Table 2 shows sensitivities and specificities for the most frequently detected microorganisms.

**TABLE 2:** Sensitivity and specificity of PCR/ESI-MS compared with BC in patients with suspected sepsis^*a*^

Species	Sensitivity [no. PCR/ESI-MS^+^/no. BC^+^ in % (ratio)]	Specificity [no. PCR/ESI-MS^−^/no. BC^−^ in % (ratio)]
Gram-positive bacteria		
* * Staphylococcus aureus	70 (19/27)	99.2 (1,421/1,433)
* * Coagulase-negative staphylococci	11 (5/45)	99.6 (1,410/1,415)
* * Streptococcus mitis/Streptococcus pneumoniae	58 (7/12)	99.4 (1,440/1,448)
* * Streptococcus pyogenes	100 (4/4)	99.9 (1,455/1,456)
* Streptococcus* species	27 (3/11)	99.7 (1,445/1,449)
* * Enterococcus faecalis	57 (4/7)	99.7 (1,449/1,453)
* * Enterococcus faecium	100 (6/6)	98.8 (1,437/1,454)
* Micrococcus* species	0 (0/1)	99.6 (1,453/1,459)
* * Cutibacterium acnes	0 (0/1)	98.8 (1,442/1,459)
* Nocardia* species	0 (0/0)	98.9 (1,444/1,460)

Gram-negative bacteria		
* * Escherichia coli	93 (50/54)	94.4 (1,327/1,406)
* * Klebsiella pneumoniae	80 (20/25)	98.3 (1,411/1,435)
* * Klebsiella oxytoca	100 (4/4)	99.9 (1,454/1,456)
* * Enterobacter cloacae complex	80 (4/5)	99.0 (1,441/1,455)
* * Pseudomonas aeruginosa	100 (5/5)	99.7 (1,451/1,455)
* * Citrobacter freundii	0 (0/0)	99.6 (1,454/1,460)
* * Serratia marcescens	62 (5/8)	99.9 (1,451/1,452)
* * Haemophilus influenzae	0 (0/2)	99.7 (1,453/1,458)
* * Bacteroides fragilis*/*Bacteroides thetaiotaomicron	50 (1/2)	99.3 (1,448/1,458)
* * Fusobacterium nucleatum	0 (0/0)	99.6 (1,454/1,460)

*Candida* species		
* * Candida albicans	67 (2/3)	99.4 (1,448/1,457)
* * Candida glabrata	0 (0/0)	99.6 (1,454/1,460)

a Species with more than 5 positive results from PCR/ESI-MS and/or BC are included.

### Results in patients with and without antimicrobial medication prior to enrollment.

**FIG 5:**
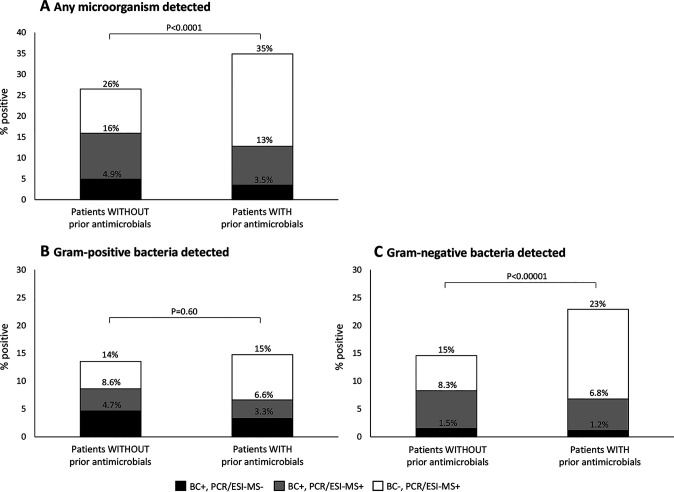
Proportion of cases positive by BC and/or PCR/ESI-MS in patients without (*n* = 857) and with (*n* = 603) prior antimicrobial treatment, overall (A) and broken down by detection of Gram-positive (B) and Gram-negative (C) organisms.

Figure 5 shows the results of BC and PCR/ESI-MS for patients without and with any prior antimicrobial medication. The BC positivity rate tended to be lower for patients with prior antimicrobial medication (13%; 77/603) than for those without prior antimicrobials (16%; 136/857) (*P* = 0.099) (Fig. 5A). However, patients treated with prior antimicrobials had significantly higher BC positivity and PCR/ESI-MS positivity combined for any microorganism (Fig. 5A) and for Gram-negative bacteria (Fig. 5C), but not for Gram-positive bacteria (Fig. 5B), than patients without prior treatment. Accordingly, the PCR/ESI-MS rate was significantly higher for patients with than for patients without prior antimicrobials, for any microorganism (*P* < 0.0001) and for Gram-negative bacteria (*P* < 0.0001), but not for Gram-positive bacteria (*P* = 16).

**FIG 6:**
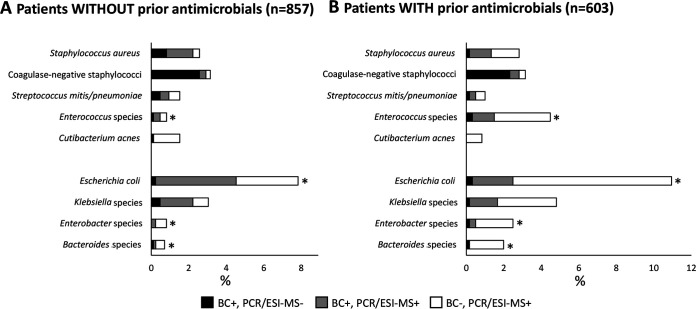
Proportions of patients without (A) and with (B) any prior antimicrobials and positive for major individual bacteria. An asterisk indicates significant (*P* < 0.05) differences regarding total proportion of positives (BC and/or PCR/ESI-MS) between cases without and with prior antimicrobials.

Figure 6 presents the detection rates of individual microorganisms in patients with and without prior antimicrobial medication. E. coli was clearly the most commonly detected microorganism in both categories. This bacterium, as well as *Enterococcus* species, *Enterobacter* species, and *Bacteroides* species, was significantly more often detected by BC and/or PCR/ESI-MS in patients who received treatment than in patients without prior antimicrobial medication (*P* < 0.05 in all cases) (Fig. 6). These organisms were also more often detected by PCR/ESI-MS alone (*P* < 0.05 in all cases).

**FIG 7:**
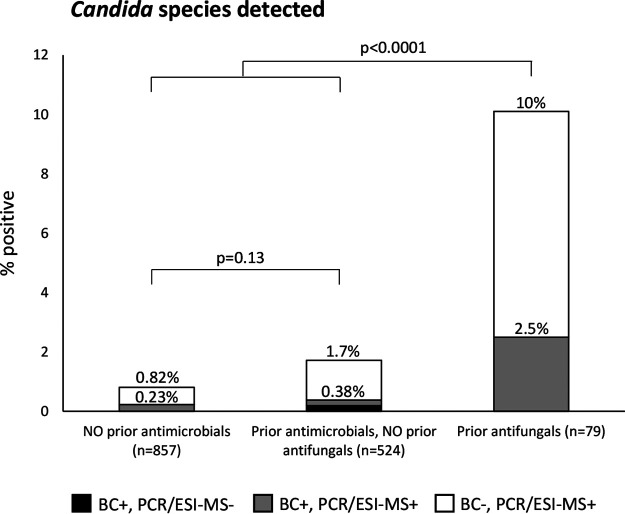
Proportion of patients positive for *Candida* species by BC and/or PCR/ESI-MS, in relation to prior antimicrobial medication.

Figure 7 shows that prior antifungal medication was strongly associated with PCR/ESI-MS positivity for *Candida* species. Prior antifungal medication was also strongly associated with BC positivity for *Candida* species: 2.5% versus 0.29% (*P* = 0.038).

### Semiquantitative results for Staphylococcus aureus and Escherichia coli DNA.

**FIG 8:**
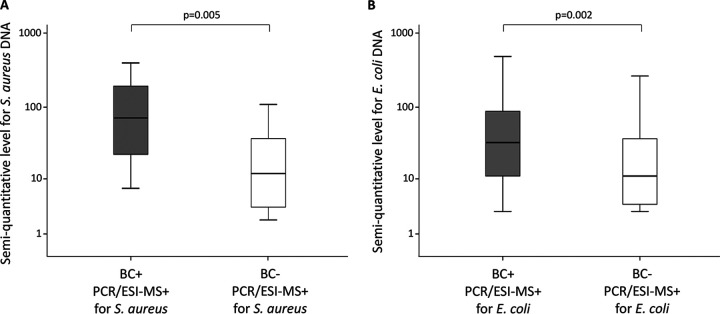
Semiquantitative levels for Staphylococcus aureus DNA (A) and Escherichia coli DNA (B) related to BC results in patients with PCR/ESI-MS positive for S. aureus and E. coli.

The semiquantitative levels of S. aureus and E. coli DNA produced by the PCR/ESI-MS system were studied as one representative each for Gram-positive and Gram-negative microorganisms. The levels were significantly higher for BC-positive PCR/ESI-MS-positive results than for BC-negative PCR/ESI-MS-positive results for both microorganisms (Fig. 8).

### Resistance markers in clinical samples.

Among 1,460 study patients, PCR/ESI-MS detected resistance markers combined with relevant bacteria in 29 cases, i.e., *mecA* in 18 cases and *vanA* in 11 cases. No patient was PCR/ESI-MS positive for *vanB* or *bla*_KPC_ together with relevant bacterial species.

The *mecA*-positive patients were PCR/ESI-MS positive for S. aureus in 10 cases and PCR/ESI-MS positive for CoNS in 8 cases. Among 10 patients who were PCR/ESI-MS positive for S. aureus and *mecA*, BC was positive for S. aureus in 7 cases, including 3 cases with MRSA and 4 cases of methicillin-susceptible S. aureus.

Among 11 patients who were PCR/ESI-MS positive for *vanA* and E. faecium, two were BC positive for E. faecium, one with VRE and one with vancomycin-susceptible E. faecium.

*bla*_KPC_ was identified by standard laboratory methods in a BC isolate of K. pneumoniae. The corresponding patient´s whole-blood sample was PCR/ESI-MS positive for K. pneumoniae but PCR/ESI-MS negative for *bla*_KPC_.

## DISCUSSION

This is the largest study of PCR/ESI-MS performed on either contrived samples or clinical samples. The study showed excellent results of PCR/ESI-MS performed on contrived samples, with very few false-negative or false-positive results. Evaluation of whole-blood samples from patients with suspected sepsis found that PCR/ESI-MS was more often positive than BC, and that trend was more pronounced in those who had received prior antimicrobial medication. While the specificity of PCR/ESI-MS was high relative to that of BC, sensitivities varied between species but were generally higher for Gram-negative than for Gram-positive bacteria.

It is well known that the BC positivity rate in a sepsis population increases with the number of BC bottles analyzed. Thus, the fact that the patients with one set of BC bottles had almost as high BC positivity rates as patients with two sets of BC bottles (Fig. 2B) indicates that the population with one set may have had a higher rate of true bloodstream infection. A higher frequency of bloodstream infection could be a reason for the high rate of PCR/ESI-MS-positive results among patients for whom one BC bottle set was collected (Fig. 2B). An alternative possible reason could be more false positives in this patient group. The obvious difference between those with one and two BC sets suggests a possibly biased or unrepresentative sample population.

The low rate of PCR/ESI-MS positivity for CoNS in the study (Fig. 4) was unexpected. However, it could perhaps be explained by the high LOD of PCR/ESI-MS for CoNS (Table S1).

Seven previous studies of PCR/ESI-MS on 5-ml whole-blood samples (9, 10, 14–18) reported BC positivity rates of 5.4 to 34% and PCR/ESI-MS positivity rates of 10.6 to 37% (19). Higher rates of PCR/ESI-MS-positive results (versus BC) were seen in 5 of those studies (9, 10, 14, 16, 18), and more BC-positive results (versus PCR/ESI-MS) were seen in 2 studies (15, 17). In the largest previous study (*n* = 616), by Vincent et al. (10), BC was positive in 11% and PCR/ESI-MS was positive in 37% of the cases. In the present study, BC was positive in 14.6% and PCR/ESI-MS was positive in 25.6% of patients.

An interesting finding of the present study was that although the BC positivity rates for Gram-positive and Gram-negative bacteria were similar (7.8% and 7.7%), the PCR/ESI-MS positivity rate was significantly higher for Gram-negative than for Gram-positive bacteria (Fig. 3). Accordingly, the sensitivity of PCR/ESI-MS compared to BC was higher for Gram-negative organisms (78%) than for Gram-positive organisms (58%). The reason for this difference is not clear. A possible explanation could be different loads of bacterial DNA in the bloodstream during sepsis. However, the PCR/ESI-MS semiquantitative levels did not differ significantly between Gram-negative and Gram-positive bacteria, as illustrated by data for S. aureus and E. coli in Fig. 8. Similarly, Ziegler et al. (20) found comparable PCR cycle thresholds of bacterial DNA in whole blood from patients with Gram-positive and Gram-negative bloodstream infection, using the LightCycler SeptiFast test. Another possible explanation could be different LOD. However, the present study could not find any general difference in LOD between Gram-positive and Gram-negative organisms (Table S1). Thus, the reason for the difference remains unclear. An interesting finding, however, was that the difference between Gram-negative and Gram-positive detections with PCR/ESI-MS was predominantly noted for patients who had received prior antimicrobial medication (Fig. 5B and C).

The design of this study enabled analysis of the importance of prior antimicrobial medication for the results of BC and PCR/ESI-MS. Similar to previous studies (21, 22), patients with prior antimicrobial medication tended to have lower BC positivity rates than patients who had not received antimicrobials (Fig. 5A). However, PCR/ESI-MS was significantly more often positive for patients with than patients without antimicrobials, and consequently, the combined results of BC and PCR/ESI-MS were more often positive for patients with than patients without prior antimicrobials (Fig. 5A). This pattern was pronounced for Gram-negative bacteria (Fig. 5C) but was not noted for Gram-positive bacteria (Fig. 5B).

Notably, differences between individual Gram-negative species were observed (Fig. 6). Prior antimicrobial medication was associated with higher combined positivity rates (BC and PCR/ESI-MS) for E. coli, *Enterobacter* species, and *Bacteroides* species, but not for *Klebsiella* species. Among Gram-positive organisms, *Enterococcus* species was more common in patients with prior antimicrobials (Fig. 6). This was an unexpected pattern, as we expected patients with prior antimicrobials to have lower PCR/ESI-MS positivity rates, in line with lower BC positivity rates. The reason for this pattern is not known. It could perhaps reflect the fact that the cases with and without prior antimicrobials represent different patient populations. Prior antimicrobial medication is, based on clinical practice patterns, likely associated with an increased likelihood of true infection and with inpatient care prior to enrollment. Bloodstream infections with *Enterobacter* species and *Enterococcus* species have been associated with long hospital durations prior to onset (23). However, the unexpected pattern of more PCR/ESI-MS-positive results in patients with prior antimicrobials could also be caused by false-positive PCR/ESI-MS results, possibly due to contamination during the extraction step, which could perhaps be more problematic with Gram-negative species.

*Candida* DNA was detected by PCR/ESI-MS significantly more often than *Candida* species was detected by BC (Fig. 4), similar to the performance of the commercial T2Candida test (T2 Biosystems, Lexington, MA ) (24). Detection of *Candida* species by both BC and PCR/ESI-MS was linked to prior antifungal medication (Fig. 7). As antifungal medication is usually based on microbiological findings and/or clinical suspicion of fungal infection, this link can reasonably be interpreted as support for BC-negative PCR/ESI-MS-positive results for *Candida* species, which would have important value for decisions in clinical practice.

A very important question is whether BC-negative PCR/ESI-MS-positive results represent true infections. It should be noted that in the present study, 2.8% of the negative contrived samples were false positive by PCR/ESI-MS. This could represent contamination or false positivity due to nonmicrobial components or microbial cell-free DNA within the blood (25). Unfortunately, the present study was not designed to evaluate the clinical relevance of BC-negative PCR/ESI-MS-positive results, as clinical data apart from SIRS data were not collected. However, there are some important findings from previous studies that should be mentioned. Jordana-Lluch et al. (26) identified 80 BC-negative PCR/ESI-MS-positive microorganisms, of which 41 microorganisms (51%) correlated with clinical findings. In another study, the same group (16) identified 84 BC-negative PCR/ESI-MS-positive microorganisms, of which 42 microorganisms (50%) had support from clinical findings. In a European ICU sepsis study (27) (a subgroup study of that described in reference 10), the 28-day mortality was found to be higher in patients with BC-negative PCR/ESI-MS-positive results than in patients with BC-negative PCR/ESI-MS-negative results (42% versus 26%; *P* = 0.001). This association with disease severity may perhaps be due to true bloodstream infection in a substantial proportion of cases with BC-negative PCR/ESI-MS-positive results. Proper evaluation of the clinical relevance of BC-negative PCR/ESI-MS-positive results requires additional studies designed to evaluate PCR/ESI-MS with detailed clinical data and should include severely ill patients without infections.

The present study’s design, with BC as the reference standard, enabled proper analysis of diagnostic sensitivity. Overall, the sensitivity of PCR/ESI-MS was 71%, which is similar to the pooled sensitivity of 66% that was recently found in the meta-analysis by Huang et al. (28). This suboptimal clinical sensitivity combined with the low frequency of false-negative results among contrived specimens indicates that a substantial proportion of patients with bloodstream infection may have bloodstream concentrations of microorganisms below the LOD for those microorganisms. The 5 species for which sensitivities were highest compared with BC in the present study, i.e., Streptococcus pyogenes, E. faecium, E. coli, Klebsiella oxytoca, and Pseudomonas aeruginosa (sensitivities > 92%) (Table 2), all had low PCR/ESI-MS LOD (8 to 16 CFU/ml [Table S1]). As it has been reported that patients with bloodstream infection may have as few as 1 to 10 CFU of circulating microorganisms per ml (25), the LOD of PCR/ESI-MS may not be clinically optimal for many microorganisms. Accordingly, the concentrations of microorganisms used in the contrived specimens of the present study may have been too high to mimic clinically relevant concentrations. Thus, due to the suboptimal sensitivity, PCR/ESI-MS cannot be used to rule out bloodstream infection.

A disadvantage of PCR/ESI-MS and other molecular methods is the limited information provided about antimicrobial susceptibility. The IRIDICA PCR/ESI-MS panel contains only four resistance markers (*mecA*, *vanA*, *vanB*, and *bla*_KPC_). However, on contrived whole blood samples spiked with microorganisms with known presence or absence of resistance in the present study, PCR/ESI-MS showed excellent performance regarding the resistance markers. Clinical samples from 10 patients were PCR/ESI-MS positive for S. aureus and *mecA*, but only 3 of them had culture-proven MRSA in their bloodstream. Similarly, 11 patients were PCR/ESI-MS positive for E. faecium and *vanA*, but only one of them had culture-proven VRE in the bloodstream. These results were not conclusive, as we do not have any additional microbiological data on the patients apart from BC and PCR/ESI-MS. Thus, there is a need for additional evaluations and, in particular, a need for new sensitive methods to determine antimicrobial susceptibility.

At the end of 2014, the PCR/ESI-MS IRIDICA BAC BSI Assay was CE marked and became commercially available for *in vitro* diagnostics in Europe. When it was used in routine practice in addition to BC at Karolinska University Hospital (29), it detected BC-negative PCR/ESI-MS-positive microorganisms that were considered clinically relevant (30). However, in April 2017, Abbott withdrew their application to the FDA regarding the IRIDICA BAC BSI assay and ceased producing IRIDICA instruments and IRIDICA test kits (29). Since then, PCR/ESI-MS has not been commercially available. Still, the present study and previous PCR/ESI-MS studies (9, 10, 16, 18) show that for patients with suspected sepsis, bacterial DNA is detected in blood more often than viable bacteria are detected by BC, especially in patients who were previously treated. This is encouraging and supports the value of further advancing new molecular diagnostics for clinical practice, which is critical for improved detection of bloodstream microorganisms and has important downstream implications for improved patient outcomes (5). Such development is further motivated by the WHO resolution on sepsis (1) and their global action plan on antimicrobial resistance (31).

The present study has several strengths. First, the large number of contrived samples spiked with microorganisms of different concentrations enabled solid conclusions regarding the analytic performance of PCR/ESI-MS. Second, the large number of clinical samples enabled comparisons between Gram-positive and Gram-negative bacteria and performance analysis with regard to individual microorganisms. Third, data on prior antimicrobial medication were collected shortly after enrollment, enabling a comparison between patients with and without prior antimicrobial treatment. Altogether, the study provided new knowledge about bacterial DNA in the bloodstream of patients with suspected sepsis.

The study also has limitations. First, the patient population was heterogenous, as the patients were enrolled at different clinical sites and were not consecutively enrolled. This design may have allowed bias between variables and may have caused lack of replicability. In addition, due to the lack of a homogenous population, we could not evaluate the additive value of PCR/ESI-MS for the etiologic spectrum of sepsis. Second, the lack of standardized blood culturing may have introduced variability regarding blood culture results. Third, standard microbiological tests apart from BC were not registered, and thus, we could not properly evaluate BC-negative PCR/ESI-MS-positive findings. Fourth, severity data apart from SIRS criteria were not registered, and thus we could not stratify patients according to the sepsis-3 classification. However, all patients had suspected sepsis according to the sepsis-2 definition, with suspected bloodstream infection and at least 2 SIRS criteria.

In conclusion, PCR/ESI-MS showed excellent performance on contrived whole blood samples. On clinical samples, it showed high specificities, moderately high sensitivities for Gram-negative bacteria and *Candida* species, and elevated positivity rates during antimicrobial treatment. These promising results encourage further development of molecular diagnostics to be used with whole blood for detection of bloodstream microorganisms in sepsis.

## Supplementary Material

Supplemental file 1

Supplemental file 2

Supplemental file 3
