# Supplementary material for: Performance of PCR/Electrospray Ionization-Mass Spectrometry on Whole Blood for Detection of Bloodstream Microorganisms in Patients with Suspected Sepsis
Source: J Clin Microbiol. 2020 Aug 24;58(9):e01860-19. doi: 10.1128/JCM.01860-19 (PMC7448645; doi:10.1128/JCM.01860-19)
Supplement: Supplemental file 1 [file JCM.01860-19-s0001.pdf]

TABLE S1 Analytical limit of detection (LOD) of PCR/ESI-MS for different microorganisms based on spiked EDTA whole blood samples. The LODs were confirmed on 20 samples per microorganism, in which a detection rate of at least 95% (minimum of 19/20 valid replicates) was required for a confirmed LOD

**Microorganism**	**Confirmed LOD CFU/mL**
**Gram-positive bacteria**	
Bacillus cereus complex	16
Clostridium perfringens	0.5
Corynebacterium diphtheriae	128
Corynebacterium jeikeium	16
Enterococcus faecalis	32
Enterococcus faecium	16
Enterococcus gallinarum	16
Listeria monocytogenes	128
Micrococcus luteus	2
Nocardia farcinica	64
Cutibacterium acnes	256
Staphylococcus aureus	32
Staphylococcus caprae	256
Staphylococcus epidermidis	256
Staphylococcus haemolyticus	128
Staphylococcus hominis	256
Staphylococcus lugdunensis	128
Streptococcus agalactiae	32
Streptococcus mitis	64
Streptococcus pyogenes	8
**Gram-negative bacteria**	
Acinetobacter baumannii	32
Acinetobacter calcoaceticus	128
Acinetobacter lwoffii	64
Bacteroides fragilis	256
Bacteroides thetaiotaomicron	64
Citrobacter freundii	64
Cronobacter sakazakii	32

Enterobacter aerogenes	4
Enterobacter cloacae complex	64
Escherichia coli	8
Fusobacterium nucleatum	8
Haemophilus influenzae	32
Klebsiella oxytoca	16
Klebsiella pneumoniae	64
Moraxella catarrhalis	64
Morganella morganii	16
Neisseria meningitidis	16
Proteus mirabilis	16
Providencia stuartii	64
Pseudomonas aeruginosa	8
Pseudomonas fluorescens	16
Salmonella enterica	64
Serratia marcescens	32
Stenotrophomonas maltophilia	4
**Mycobacteria**	
Mycobacterium fortuitum	256
***Candida* species**	
Candida albicans	8
Candida dubliniensis	4
Candida glabrata	16
Candida parapsilosis	8
Candida tropicalis	8
