# Supplementary material for: Performance of PCR/Electrospray Ionization-Mass Spectrometry on Whole Blood for Detection of Bloodstream Microorganisms in Patients with Suspected Sepsis
Source: J Clin Microbiol. 2020 Aug 24;58(9):e01860-19. doi: 10.1128/JCM.01860-19 (PMC7448645; doi:10.1128/JCM.01860-19)
Supplement: Supplemental file 2 [file JCM.01860-19-s0002.pdf]

Table S2 Performance of PCR/ESI-MS on 2,500 positive contrived specimens

Contrived organism	Organism present		Organism NOT present	
	**PCR/ESI-MS+**	**PCR/ESI-MS-**	**PCR/ESI-MS+**	**PCR/ESI-MS-**
Acinetobacter baumannii	49	1	0	2,450
Acinetobacter calcoaceticus	50	0	1	2,449
Acinetobacter lwoffii	49	1	0	2,450
Bacillus cereus complex	50	0	0	2,450
Bacteroides fragilis	49	1	0	2,450
Bacteroides thetaiotaomicron	50	0	0	2,450
Candida albicans	49	1	0	2,450
Candida dubliniensis	49	1	0	2,450
Candida glabrata	50	0	0	2,450
Candida parapsilosis	50	0	0	2,450
Candida tropicalis	50	0	2	2,448
Citrobacter freundii	49	1	0	2,450
Clostridium perfringens	50	0	0	2,450
Corynebacterium diphtheriae	50	0	0	2,450
Corynebacterium jeikeium	48	2	0	2,450
Cronobacter sakazakii	50	0	0	2,450
Enterobacter aerogenes	50	0	1	2,449
Enterobacter cloacae complex	50	0	0	2,450
Enterococcus faecalis	50	0	0	2,450
Enterococcus faecium	49	1	0	2,450
Enterococcus gallinarum	50	0	0	2,450
Escherichia coli	49	1	3	2,447
Fusobacterium nucleatum	49	1	1	2,449
Haemophilus influenzae	48	2	0	2,450
Klebsiella oxytoca	50	0	0	2,450
Klebsiella pneumoniae	50	0	2	2,448
Listeria monocytogenes	49	1	0	2,450
Micrococcus luteus	49	1	2	2,448
Moraxella catarrhalis	50	0	0	2,450
Morganella morganii	49	1	0	2,450
Mycobacterium fortuitum	49	1	0	2,450
Neisseria meningitidis	49	1	1	2,449

Nocardia farcinica	50	0	4	2,446
Cutibacterium acnes	50	0	7	2,443
Proteus mirabilis	48	2	0	2,450
Providencia stuartii	50	0	0	2,450
Pseudomonas aeruginosa	50	0	0	2,450
Pseudomonas fluorescens	50	0	1	2,449
Salmonella enterica	50	0	0	2,450
Serratia marcescens	50	0	0	2,450
Staphylococcus aureus	50	0	3	2,447
Staphylococcus caprae	50	0	0	2,450
Staphylococcus epidermidis	49	1	3	2,447
Staphylococcus haemolyticus	50	0	0	2,450
Staphylococcus hominis	49	1	2	2,448
Staphylococcus lugdunensis	50	0	0	2,450
Stenotrophomonas maltophilia	50	0	0	2,450
Streptococcus agalactiae	50	0	0	2,450
Streptococcus mitis	49	1	0	2,450
Streptococcus pyogenes	50	0	0	2,450
