# Supplementary material for: Performance of PCR/Electrospray Ionization-Mass Spectrometry on Whole Blood for Detection of Bloodstream Microorganisms in Patients with Suspected Sepsis
Source: J Clin Microbiol. 2020 Aug 24;58(9):e01860-19. doi: 10.1128/JCM.01860-19 (PMC7448645; doi:10.1128/JCM.01860-19)
Supplement: Supplemental file 3 [file JCM.01860-19-s0003.pdf]

TABLE S3 Combined results of blood culture and PCR/ESI-MS on whole blood from 1,460 patients

Organisms	Blood culture +		Blood culture -	
	PCR/ESI-MS+	PCR/ESI-MS-	PCR/ESI-MS+	PCR/ESI-MS-
**Gram-positive bacteria**				
Staphylococcus aureus	19	8	12	1,421
Enterococcus faecium	6	0	17	1,437
Staphylococcus epidermidis	5	15	3	1,437
Streptococcus mitis/pneumoniae	7	5	8	1,440
Cutibacterium acnes	0	1	17	1,442
Nocardia species	0	0	16	1,444
Streptococcus species	3	8	4	1,445
Coagulase-negative staphylococci	0	14	0	1,446
Enterococcus faecalis	4	3	4	1,449
Micrococcus species	0	1	6	1,453
Staphylococcus hominis	0	6	1	1,453
Streptococcus pyogenes	4	0	1	1,455
Staphylococcus capitis	0	5	0	1,455
Streptococcus dysgalactiae	1	0	1	1,458
Clostridium perfringens	0	0	2	1,458
Clostridium septicum/tertium	0	1	1	1,458
Gemella morbillorum	0	1	1	1,458
Peptostreptococcus anaerobius	0	2	0	1,458
Aerococcus urinae	1	0	0	1,459
Aerococcus viridans	0	0	1	1,459
Anaerococcus tetradius	0	0	1	1,459
Bifidobacterium breve	0	0	1	1,459
Bifidobacterium longum	0	0	1	1,459
Clostridium botulinum	0	0	1	1,459
Kocuria rhizophila	0	0	1	1,459
Staphylococcus haemolyticus	0	0	1	1,459
Streptococcus intermedius	0	0	1	1,459
Streptococcus vestibularis	0	0	1	1,459
Clostridium species	0	1	0	1,459
Eggerthella lenta	0	1	0	1,459
**Gram-negative bacteria**				
Escherichia coli	50	4	79	1,327
Klebsiella pneumoniae	20	5	24	1,411
Enterobacter cloacae complex	4	1	14	1,441

Bacteroides fragilis/thetaiotaomicron	1	1	10	1,448
Pseudomonas aeruginosa	5	0	4	1,451
Serratia marcescens	5	3	1	1,451
Haemophilus influenzae	0	2	5	1,453
Klebsiella oxytoca	4	0	2	1,454
Citrobacter freundii	0	0	6	1,454
Fusobacterium nucleatum	0	0	6	1,454
Proteus mirabilis	1	1	2	1,456
Stenotrophomonas maltophilia	1	0	2	1,457
Morganella morganii	1	2	0	1,457
Bacteroides caccae	0	0	3	1,457
Fusobacterium necrophorum	1	0	1	1,458
Aggregatibacter segnis	0	0	2	1,458
Bacteroides uniformis	0	0	2	1,458
Bacteroides vulgatus	0	0	2	1,458
Enterobacter aerogenes	0	0	2	1,458
Haemophilus parasuis	0	0	2	1,458
Odoribacter splanchnicus	0	0	2	1,458
Shigella boydii	0	0	2	1,458
Acinetobacter baumannii	0	1	1	1,458
Citrobacter braakii	1	0	0	1,459
Acinetobacter lwoffii	0	0	1	1,459
Aeromonas eucrenophila	0	0	1	1,459
Aeromonas hydrophila	0	0	1	1,459
Aeromonas jandaei/veronii	0	0	1	1,459
Aeromonas salmonicida	0	0	1	1,459
Bacteroides ovatus	0	0	1	1,459
Bordetella avium	0	0	1	1,459
Bordetella bronchiseptica/parapertussis	0	0	1	1,459
Bordetella petrii	0	0	1	1,459
Citrobacter youngae	0	0	1	1,459
Enterobacter cowanii	0	0	1	1,459
Escherichia vulneris	0	0	1	1,459
Ewingella americana/Pantoea agglomerans	0	0	1	1,459
Haemophilus parainfluenzae	0	0	1	1,459
Legionella pneumophila	0	0	1	1,459
Neisseria gonorrhoeae	0	0	1	1,459
Neisseria meningitidis	0	0	1	1,459
Ochrobactrum anthropi	0	0	1	1,459

Prevotella denticola	0	0	1	1,459
Salmonella enterica	0	0	1	1,459
Tatumella ptyseos	0	0	1	1,459
Bacteroides species	0	1	0	1,459
Moraxella species	0	1	0	1,459
Oligella ureolytica/urethralis	0	1	0	1,459
Pasteurella multocida	0	1	0	1,459
Proteus vulgaris	0	1	0	1,459
Salmonella species	0	1	0	1,459
**Mycobacteria**				
Mycobacterium species	0	0	5	1,455
**Candida species**				
Candida albicans	2	1	9	1,448
Candida glabrata	0	0	6	1,454
Candida species	1	0	1	1,458
Candida parapsilosis	1	0	1	1,458
Candida tropicalis	1	0	1	1,458
Candida dubliniensis	0	0	2	1,458
Candida famata	0	0	1	1,459
**Total positives**	**149**	**99**	**324**	
